# Disutility Study for Adult Patients with Moderate to Severe Crohn’s Disease

**DOI:** 10.36469/9685

**Published:** 2019-03-26

**Authors:** Melany Worbes-Cerezo, Beenish Nafees, Andrew Lloyd, Katy Gallop, Imran Ladha, Cicely Kerr

**Affiliations:** 1 Janssen-Cilag Ltd, UK; 2 Nafees Consulting Limited, London, UK; 3 Acaster Lloyd Consulting, London UK; 4 The author was employed by Janssen-Cilag Ltd at the time of the study

**Keywords:** Crohn’s disease, utility, time trade-off, health-related quality of life, adverse events, surgical complications

## Abstract

**Background:**

Crohn’s disease (CD) treatments and associated adverse events (AEs) can be burdensome for patients. However, specific values which quantify the impact on health-related quality of life (HRQL) for economic evaluation are lacking.

**Objectives:**

This study aimed to elicit health utility values for AEs related to biologic treatment and surgical complications for CD in the UK.

**Methods:**

Health states were developed by literature review and interviews with CD patients (n=6) and gastroenterologists (n=3). Draft health states were validated in cognitive debrief interviews with patients (n=4) and gastroenterologists (n=2). Treatment AEs were described with moderate-severe CD (reference state) and included hypersensitivity, injection site reactions, serious infection, lymphoma, and tuberculosis. Surgical complications were described following bowel surgery (reference state) and included anastomotic leak, wound infection, prolonged ileus/bowel obstruction, and intra-abdominal abscess. Health states were valued by 100 members of the general public who completed background questions, EQ-5D-3L, visual analogue scale rating task and time trade-off (TTO) interviews.

**Results:**

The mean TTO value for reference states ‘moderate to severe CD’ and ‘bowel surgery’ were 0.70 (SD=0.28) and 0.69 (SD=0.28). Participants rated lymphoma as the worst AE/surgical complication state (0.44, SD=0.37), followed by tuberculosis (0.47, SD=0.85) and anastomotic leak (0.48, SD=0.38). Values of other AE/surgical complication states ranged from 0.76 (hypersensitivity) to 0.56 (intra-abdominal abscess).

**Conclusions:**

This study provides utility estimates for AE and surgical complication health states not previously assessed in the context of CD. As new treatments are emerging, it is important to include these influences on quality of life in any economic evaluation of treatments.

## Background

Crohn’s disease (CD) is a chronic inflammatory bowel disease and currently affects at least 115 000 people in the UK.^1^,^2^ Symptoms of active CD include diarrhea, abdominal pain, and gastrointestinal bleeding, and this can have a substantial impact on patients’ health-related quality of life (HRQL).^3^ Although disease activity is a good predictor of HRQL, people who are asymptomatic can also report reduced HRQL, higher anxiety about risks and AEs, and worse coping than other bowel conditions such as ulcerative colitis.^4^ Up to a third of people with CD are diagnosed before age of 21 years however there is lack of research on treatments for the young population.^5^,^6^ This can be particularly problematic as this is the time when young adults are deciding upon their career, education and other goals.

Conventional treatments for CD include systemic immunosuppressants and corticosteroids.^6^–^9^ Moderate or severe patients are commonly treated with biologic therapies such as anti-tumor necrosis factor-alpha (TNFα) antibodies which have been proven as clinically effective,^7^,^8^,^10^ and also show improvement in HRQL amongst some CD patients.^11^ New treatments continue to emerge for this condition which offer a reduction of the symptoms of the disease but also can be associated with adverse events (AEs) which need to be considered in any decision-making context.^12^ In some cases, patients do not respond to biologics and the ongoing disease process can continue to damage the bowel despite the best available therapies. Such patients may require bowel resection, which is a burdensome procedure and is also associated with complications, comorbidities and significant recovery period.^13^ Modern treatments such as biologics have been shown to delay the need for bowel surgery, which is a substantial area of additional benefit for these patients.^13^

When a new treatment emerges for CD there are many issues that need to be considered to fully understand its efficacy. While drug regulators focus on safety and efficacy, many reimbursement bodies or payers are also interested in effectiveness of the drug in clinical practice and the likely longer term benefits which are commonly not observed in clinical trials.^8^,^10^ The National Institute for Health and Care Excellence (NICE) in England, the Scottish Medicines Consortium (SMC) in Scotland and several other Health Technology Assessment (HTA) bodies in Europe have a strong focus on understanding the health gain associated with a drug intervention as well as the likely cost-effectiveness of the intervention taking into account impact of AEs and complications as well as direct health benefit of the drug.^6^ NICE and others use HRQL data as a key component of economic evaluation of cost-effectiveness for the estimation of quality adjusted life years (QALYs). Clinical trials can describe the full HRQL impact of novel therapies in a given period of time. However, the specific HRQL impact of AEs and long-term consequences such as bowel surgery and its subsequent complications are often difficult to capture in this context. The present study was designed to capture these missing data elements by estimating utilities for AEs related to biologic treatment and complications related to surgery for CD in the UK.

## Methods

The study was divided into two parts: the first part of the study involved a literature review and qualitative patient and clinician interviews; the results from these were used to further select and confirm health states and develop health state descriptions (health states development). The second part of the study involved the valuation of the health states by members of the general public in the UK (main study) as methods requirements for HTA bodies such as NICE in England requires utility values that incorporate general public perspectives for the estimation of QALYs in economic analysis.^6^ The study protocol was reviewed and approved by an independent review board: Salus IRB (date of approval: 2nd June 2016).

### Health States Development

#### i. Literature Review

A search strategy was developed to identify articles describing the burden of CD and its treatment on HRQL, particularly focusing on biologic treatment-related AEs and surgery-related complications in the last ten years. Searches were conducted in Embase and Medline originally in March 2016 and updated in 2018. Search terms included symptoms, HRQL, treatment, and treatment-related AEs.

The initial search led to the identification of 2062 abstracts (March 2016). All abstracts were reviewed and screened and 252 were selected as relevant. Studies were excluded if they were case studies, if the sample were children, or the study did not include relevant information such as treatment-related data. Full text articles were reviewed if they included biologic treatment, conventional treatments, and/or surgery and described the impact of CD, AEs or surgery-related complications on HRQL. Seventeen papers met these criteria and were reviewed for full results.

The majority of the literature focused on reporting AEs as part of clinical trials and prevalence rates.^7^,^14^–^16^ Johnson et al (2007)^17^ conducted a preference study to assess the willingness of CD patients to accept life-threatening AE risks in exchange for symptom relief. Participants (N=580) were presented with hypothetical treatments which included features such as daily symptoms and activity limitations, serious complications (fistulas, abscesses, bowel obstructions), time between flare-ups, oral steroid use and risk of 3 serious adverse events (SAEs) known to be associated with CD treatment (progressive multifocal leukoencephalopathy (PML), serious infections, and lymphoma). The mean maximum acceptable annual risk (MAR) for each of the AEs was calculated for various levels of clinical benefit. Symptom severity was the most valued feature in treatment preferences. Higher MAR was observed for trade-off tasks involving higher levels of clinical benefit. For improvements from severe daily symptoms to remission and from moderate daily symptoms to remission, the MARs ranged from 0.69%–0.81% and from 0.39%–0.55%, respectively. The results showed that patients were willing to accept elevated risk of life-threatening AEs in exchange for treatment benefits. CD and associated treatment can also have a significant impact on patients’ emotional HRQL,^18^ and symptoms can affect patient’s level of physical activity and work.^19^,^5^ A survey of people with irritable bowel disease (IBD), including those with CD, found that 40% of participants felt that they could not have the career of their choice due to their condition, people can miss work and that IBD can potentially lead to early retirement due to its impact on daily life.^5^ A qualitative study conducted in the UK with 30 moderate to severe CD patients reported that patients had concerns which ranged from diet, lifestyle to relationships, confidence and autonomy.^20^ Wilburn et al^20^ found that patients worried about change in diet, keeping up levels of hygiene, and likelihood of getting a serious infection. However, patients reported more concerns about how CD changed their lives emotionally. Participants expressed that CD had taken away the ‘pleasure in life,’ affected their self-esteem, changed their role in life, e.g. as a provider, and affected their feeling of attractiveness and intimacy with partner. This study highlights the major emotional burden that CD can have.

#### ii. Health States Selection

A list of all AEs and surgery-related complications was compiled based on the literature review findings and summary product characteristics of biologic treatments. Of these, some common and some rare AEs of biologic treatment and surgery-related complications were selected based on the most recent NICE appraisal available at the time TA 352 “Vedolizumab for treating moderately to severely active CD after prior therapy.”^10^ Additionally AEs which were relevant to another biologic treatment (ustekinumab) were also included to inform economic evaluation in the ustekinumab HTA submission. AEs and surgical complications for which existing literature did not report utility values were also selected. The AEs and complications selected were: serious infection, injection site reactions, lymphoma, tuberculosis, wound site infection, prolonged ileus/bowel obstruction, intra-abdominal abscess, anastomotic leak, and hypersensitivity. In addition, separate health states were developed to describe moderate to severe CD only, and CD with bowel surgery.

#### iii. Interviews with Patients and Clinicians

Interview participants were recruited through a specialist recruitment panel in the UK. The sample size was based on previous studies which were similar in methodology.^35^,^36^ In these studies, the number of clinician and patient interviews have been similar and sufficient to inform health state development. Potential CD participants were asked to take part if they were 18 years of age; currently a resident of the UK; had a current diagnosis of CD; currently or previously receiving biologic treatment (infliximab, adalimumab or vedolizumab) for at least 6 months and/or had bowel surgery in the last 12 months; had experienced at least one of the AEs or complications stated, and were able to understand the study objectives and the tasks as judged by the principal investigator/and study team. The biologic treatment and bowel surgery inclusion criterion ensured that patients had current diagnosis of moderate to severe CD. Clinicians were eligible for the interviews if they were currently practicing and prescribing treatments for CD; and a resident of the UK.

Patients were only asked to describe the AEs/complications (frequency, duration, and intensity) that they had experienced whereas clinicians were asked to describe the typical impact for a patient of each of the AEs and complications listed above in detail. All participants were asked to comment on surgery, biologics and impact of treatment and the impact of the treatment-related AEs on HRQL in moderate to severe CD patients. Some examples of the findings are discussed below. All interviews were conducted by telephone using semi-structured interview guides informed by the literature review and were audio-recorded and transcribed. Patients provided written informed consent prior to the interview. The following sections summarise themes from the interviews which informed the health state descriptions that were later developed. The summary includes verbatim quotes from interviews, with anonymous individual identifier shown in brackets after each quote.

##### Symptoms of Moderate to Severe CD

Clinicians were asked to comment on the experience of symptoms of moderate to severe CD only. Clinicians (N=3) and patients (N=6) reported similar symptoms such as diarrhoea, abdominal pain, fatigue, nausea and vomiting, weight loss, and fever.

Several patients (N=4) reported experiencing tiredness or fatigue, which affected their energy levels and meant that they could not do as many activities as they could previously or needed longer to recover from physical activities:

‘My energy levels aren’t what they were. Frustrating but I learnt to deal with it and reserve energy.’ [Patient (PT) 5]

However several patients were still able to exercise regularly.

Abdominal pain and stomach cramps were reported by all participants. They described their pain as very severe which would affect their day and may require them to take strong painkillers:

‘Pain can be really painful and bring tears to my eyes.’ (PT3)

‘Cramps are like a knife in the stomach, and I double up in pain.’ (PT6)

##### Surgery

All clinicians reported that typically surgery can have an impact on mobility and physical activities for the immediate 5–7 days post-surgery however then patients should resume most activities, unless complications arise. Clinicians felt that recovery from surgery can sometimes take up to 6–8 weeks and the impact of surgery on emotional wellbeing is often underestimated.

‘People can recover from surgery pretty quickly these days. On average, people with emergency surgery will be much sicker, will be in hospital much longer, and more medication so they will take longer.. If it was elective surgery, bowel obstruction for example, they will be home usually within 3–5 days. People are encouraged to start mobilising a day or so after surgery. They can start walking, they can’t do vigorous exercise like swimming or running they gradually re-introduce their diet for the first few weeks. If people were on high levels of medication, steroids, and poorly nourished, then they may stay for longer. If they were on biologics, they are slower to recover.’ [Clinician (CL) 1]

Clinicians reported that patients can feel traumatised especially if complications occur. They also reported that patients can also fear the risk of surgery again in the future. Two patients took time off work for up to 8 weeks to recover and needed carer support to do their usual activities and self-care. Patients also felt depressed and sad during recovery, and did not want to go out.

‘I was very worried. If it was another operation it might have been easier but because it is to do with your intestines it’s harder. I contemplated seeing a counsellor because no-one understood what I was going through.’ (PT4)

##### Biologic Treatment

Clinicians discussed the rapid benefit that some patients experience when certain treatments are initiated. Some biologic treatments were considered transformative for patients. There is a positive impact on mood and emotional wellbeing because patients see improvement quickly. Those patients who were on biologic treatment reported that their symptoms and HRQL had improved after treatment.

‘The use of a biologic should improve mobility and independence. The introduction of biologics has transformed the lives of patients.’ (CL2)

‘Can see a dramatic response in 2 weeks (with biologics).’ (CL3)

##### Adverse Events and Surgical Complications

Of the AEs selected, clinicians reported that lymphoma is very rare however it would have a huge impact on patients. Lymphoma not only limits patients’ activity but also affects them emotionally. None of the patients interviewed reported experiencing lymphoma at screening or during interviews.

‘This has a huge impact. You worry you may die. You are not worried with CD but you are with lymphoma.’ (CL1)

‘Whatever the nature of the lymphoma it will have a negative impact on all areas of HRQL, could have a very profound impact if it’s an aggressive lymphoma.’ (CL2)

When patients were asked to describe if they had experienced a serious infection, they all described having bronchitis which was characterised by coughing for a few weeks, trouble breathing, and requiring hospitalisation. The infection had a significant impact on their social life, limited their going out due to feeling very tired.

Clinicians described a serious infection such as pneumonia as very common for patients. It could lead to hospitalisation, requiring antibiotics and care. Clinicians reported that a serious infection would impact a patient’s daily life as they would feel very tired, and would not be able to work, or even need help with self-care.

‘Pneumonia is most common, requires hospitalisation. If it is severe, you might be bed bound, and would need a lot of support, and with self-care’ (CL3)

Other AEs and surgical complications were described in similar detail whereby patients were asked if they had experienced each event, and describe its duration, severity, frequency and its impact on their HRQL. Clinicians were asked to describe how an average patient would experience each event/complication and its duration, severity, frequency and impact on HRQL.

#### iv. Draft health states and validation

Descriptions were drafted for each of the health states informed by the interviews with patients and clinicians. Health states were structured into bullet points, each describing CD and associated symptoms, the side effect or complication and HRQL (physical activity and mobility, social activity, and emotional wellbeing).

**Figure 1:**
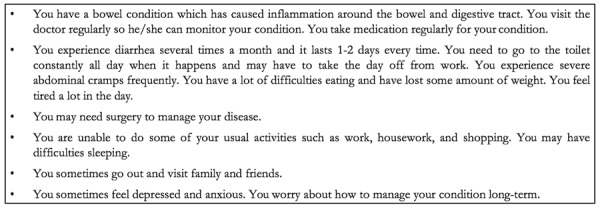
Example Health State Moderate to Severe CD (Reference State)

Cognitive debriefing interviews were conducted by telephone to validate the draft health states with new patients (N=4) and some of the same clinicians (N=2). Participants were sent the draft health states to refer to during the interview. Clinicians were asked to comment on the accuracy of the health states in describing a typical patient with each of the AEs and surgical complications; patients were asked to comment on the extent to which it reflected their experience of the AEs or complications they had experienced. Clinicians and patients were also asked to comment on the wording of the health states. Clinicians suggested changes to wording to improve the accuracy of the health states such as that patients would experience diarrhoea and tiredness more frequently than originally stated. Patients did not request any changes. Minor wording changes were suggested for other AE health states. The final heath states used for the valuation exercise are shown in Appendix 1 and example is shown in Figure 1.

### Main Study

#### v. Sample

Members of the general public were recruited from newspaper advertisements and an existing database of volunteers (10% of the total sample). Recruitment aimed to represent the general population demographics in the UK by age, and gender.^21^ Participants were eligible to take part if they were 18 years or over, currently a resident in the UK and able to understand the study requirements. Participants completed a standardised measure of health - the EQ-5D-3L,^22^ a brief socio-demographic questionnaire and the time trade-off (TTO) interview.^23^

Before the TTO exercise, all participants first ranked the health states on a scale of 0 (worst imaginable health) to 100 (full health) on a visual analogue scale (VAS). Participants were asked to read the states one at a time, including the state called ‘dead,’ and place them on this scale. This served to familiarise participants with the health states, and the VAS ranking and scores were also recorded. For the TTO exercise, participants were then asked to imagine that they were currently experiencing each health state in turn, by reading the vignette descriptions. For each health state, participants were required to choose whether they preferred either:

1. to live in the health state for a period of 10 years followed by death;
2. to live in 10-x years of full health (see below for how x is varied during the process); or
3. to indicate that the two previous options were equally desirable – no preference between the two.

The process incorporates a ‘ping-pong’ approach with the number of years in “full health” in option (2) traded back and forth between higher and lower values that iteratively narrow to the point of indifference (option 3), where the participant has no preference between the reduced number of years in “full health” (option 2) versus 10 years in a less than full heath health state (option 1). All health states were identified using only a symbol; no reference was made to the name of the health state so as not to bias the participant. The data collected for each state was in the form of a utility value ranging from 0 (worst health) to 1 (full health). Each interview lasted approximately 30–45 minutes and participants were reimbursed for their time.

### Statistical Analysis

As this study had no specific hypothesis, it was not possible to conduct a formal sample size calculation. Previous studies of societal utility values in Europe have used similar sample sizes to those included in this study.^24^

Descriptive statistics were used to summarise socio-demographic data, with means and standard deviations (SD) for continuous data and percentages for categorical data. EQ-5D data was scored according to the developer’s guidelines (EQ-5D-3L user guide). The TTO and VAS scores were analysed and calculated as means and SD with 95% confidence intervals (CI) for each health state. The TTO value of each AE/surgical complication was subtracted from the ‘moderate to severe CD’ reference state or ‘surgery’ reference state value to estimate a disutility associated with each AE/surgical complication.

## Results

### Sample Characteristics

100 general public participants were recruited across the UK. Table 1 presents the demographics for the sample overall and compared to UK census population data.^21^ The sample largely reflected the UK general population, however the study sample was slightly more diverse in employment and a high proportion of participants were in full-time employment (73%). The average age of the study sample was 36 years and 39% of the sample was male. The majority of the study sample was in good health (mean index score=0.93).

### Health Utility Values

Table 2 shows the mean TTO utilities for each of the health states and the magnitude of the disutility for each AE and complication.

**Figure 2:**
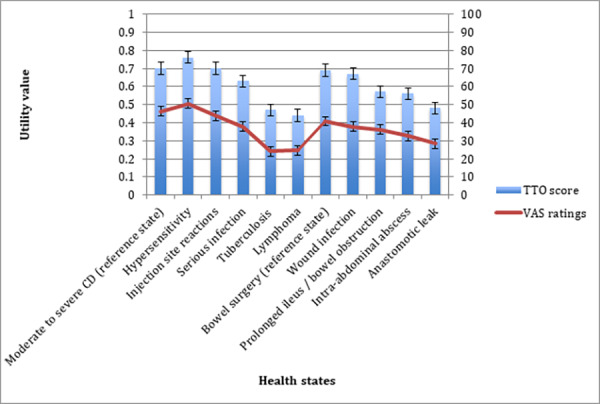
Mean and 95% CI TTO Utility Score (0–1) and VAS Rating (0–100) for each Health State (Best to Worst from Reference State)

TTO utilities for the reference health states were similar (‘moderate to severe CD’=0.70, ‘bowel surgery’=0.69). Participants rated lymphoma as the worst state (0.44), followed by tuberculosis (0.47) and anastomotic leak (0.48). Other states ranged from 0.76 (hypersensitivity) to 0.56 (intra-abdominal abscess). In general, disutilities were largest moving from the ‘moderate to severe CD’ state to lymphoma (−0.26). The VAS ranking method showed most of the states followed the same order, however tuberculosis was rated slightly lower (24.1) than lymphoma (24.7) (Figure 2).

## Discussion

The current study estimates utility values/disutilities associated with treatment for CD. The health state vignettes described AEs related to biologic treatment and surgery-related complications in moderate to severe CD patients in the UK. Health states were developed from a literature review and qualitative interviews with patients and clinicians in an iterative process of open-ended interviews and review. Once developed, a TTO methodology was employed to elicit utility values for each of the eleven health states from a broadly representative sample of the general public in the UK. These values can support economic evaluations of current and future treatments for CD whereby utility values weighted by societal preference are a requirement of HTA bodies.

This is the first study to elicit general public values for some of these AEs and complications in CD. Previous research reported the incidence rates for some of these AEs such as serious infection, and measured the impact of treatments using measures such as SF-36, the EQ-5D, IBD- Control,^25^ and IBD-Q,^26^ however utility data for the current AEs and surgery-related complications were not found.

The findings reflect the perceived burden of the CD health states from the perspective of the UK general public. The reference state – moderate to severe CD had an estimated utility score of 0.70 (95% CI=0.65–0.75) which shows that the value for the perceived burden of CD is markedly lower than the average health utility of the general public participants in this study as measured by EQ-5D-3L (0.93). This utility estimate for moderate to severe CD is consistent with values previously reported in the literature. In the NICE submission of vedolizumab,^10^ an algorithm produced by Buxton et al (2007)^27^ was used to report utilities estimated from trial datasets using a mapping function based on the relationship between the CD Activity Index (CDAI) and EQ-5D. Utility for patients with treatment response in full remission (CDAI<150) was estimated as 0.83 and for patients with mildly–moderately active disease (CDAI 150–220) as 0.69 by using the algorithm. This second value is very close to the estimate for the moderate to severe CD reference state in the current study. Huaman et al (2010)^28^ reported a mean EQ-5D-3L score for moderate-severe CD patients in Spain that was slightly higher at 0.76 (SD=0.18). The utilities reported here are from studies which used different methods of utility elicitation and therefore, although these provide useful points of reference, comparisons should be made with caution.

The study found a similar utility value for moderate to severe CD and for the post bowel surgery state. This may reflect the fact that the perceived gastrointestinal symptom burden in the two states is similar from the perspective of the general public. The surgery state describes how the patient has recently undergone bowel surgery, but also states that the patient has recovered from the procedure and returned to usual activities. Mesterton et al (2009)^29^ reported post-surgical utility of 0.77 in a sample of Swedish patients (n=420) using the 15D instrument. Marchetti et al (2013)^30^ reported 3 months post-surgery utility was 0.82 using a modelling approach. The post-surgical utility estimate from the current study is slightly lower than these scores. In addition, clinicians in the current study reported that if patients undergo surgery, they are likely to need surgery again which was included in the health state descriptions. Cohen et al (2002)^31^ and other studies all report that disease activity recurs after surgery which can impair HRQL.^32^,^33^ The surgery utility value estimated in the current study is supported by the clinician and patient interviews which reported that the burden of surgery could last for several weeks.

The estimates of utilities for AEs in the current study reflected quite a wide range of scores. Two health states (hypersensitivity and injection site reactions) were associated with no decline in utility from the moderate to severe CD reference state. In fact, the hypersensitivity state’s value was six points higher than the reference state. Clearly it is illogical that experiencing a side effect would improve quality of life or would be preferred, so it is possible this may be due to measurement error. The CIs for the base state and hypersensitivity state overlap. However, it seems more likely the difference is due to one or both of two differences from the moderate to severe CD reference state which may have made the hypersensitivity AE state more preferable. Firstly, the phrase “You may need surgery to manage your disease,” was included in the reference state but not in any of the AE health states to avoid potential double weighting of disutility associated with potential surgery if utilities for AEs are combined with the reference state utility when implemented in health economic models. Secondly, in the hypersensitivity AE state the patient was described as able to do usual activities, whereas in the moderate to severe CD reference state, the patient was unable to do some of their usual activities. This was based on clinical input that the rash and itching associated with hyspersensitivity had minimal or no impact on patients’ usual activities. This made the hypersensitivity state better than the reference state. Due to these issues, we recommend that the disutility for the hypersensitivity state be set to 0.

The disutilities for some AEs were notably large, in particular those for tuberculosis and lymphoma, reflecting the severity of these conditions. Both of these AEs were considered the worst AEs by clinicians during interviews and were described as having the most significant burden on patients. However it should be noted that both of these AEs are very rare. These were important to include in this study due to their significant HRQL impact, however they are not frequently experienced by patients. Verifying the accuracy of the utility values for AEs and surgical complications from the literature was difficult because no studies were found which had estimated them in the context of CD.

This study does have some important limitations which should be considered when reviewing the findings. Deriving utility values from health state vignettes has been criticized, partly because they are usually based on a small number of qualitative interviews to construct the vignettes. It is also difficult to confirm the content validity of the vignettes themselves beyond undertaking validation interviews with experts.^6^ The vignettes describe ‘typical’ patients in various states and so don’t reflect the heterogeneity between patients (within those who experience the same health state). In the present study, we tried to maximize their accuracy by drawing on different sources of information – literature, and qualitative research. We also aimed to estimate disutilites for the complications and AEs so that findings from this study could be combined with data from other research regarding the day to day burden of CD.^9^ In this context, the vignette methodology allows us to fill in gaps, it can be used to supplement other sources of data, for example EQ-5D utilities elicited for reference states in clinical trials or observational studies. Therefore, the most important result is the difference in utility between the reference state and the additional experience of an AE/surgical complication. The chosen AEs were challenging to characterize given the variability in presentation. Attempts were made to ensure the robustness of the health state development process in this study to establish that the description of AEs were both sufficiently accurate and detailed. However, it was not possible to capture detailed qualitative data from multiple patients who had experienced every AE or complication, so the descriptions were more reliant on input from physicians. It is important to note that current health state vignettes reflect ‘typical’ patients who are moderate to severe and actively symptomatic and not patients who may be at different stages of disease, e.g. in remission. Utilities which reflect patients at other stages may be different and applying these to other patient groups should be done with caution. In the present study, the focus was to elicit public preferences moderate to severe CD and to add to a missing base of utility data. A further concern with the vignette approach is that it requires members of the general public to imagine what it is like to experience a health state in order to value it. This is true of all societal valuation approaches, including elicitation of preference weights for generic utility measures e.g. the EQ-5D, however is arguably more challenging with more disease-specific descriptions.

Much of the potential value of this study in our view is that the project has estimated disutilities for certain adverse events related to biologic treatment and complications from bowel surgery. It is a significant challenge to prospectively measure the impact of adverse events on HRQL using a measure like EQ-5D for several reasons. When patients experience acute events they will often not feel well enough or motivated to complete questionnaires. Some events are included in economic models because they have a significant cost and/or HRQL impact (such as lymphoma in the present study) but prospectively identifying such patients is very challenging. Vignette studies have methodological limitations but offer a useful method for estimating these types of events in economic models. It is possible that AEs and complications may only have a limited impact on a cost effectiveness analysis because they often don’t last very long, affect only a small proportion of patients and may be amenable to treatment. Estimating their impact using vignette methods (as opposed to prospective EQ-5D data collection) may suffice for modelling purposes if the values have little overall effect on the cost effectiveness ratios. Wolowacz et al (2016)^34^ in their guidance paper recommend that research resources are targeted at utility data collection that will have an important effect on the final cost effectiveness analysis and that the model itself could guide such decisions. This philosophy could also be applied in the present context. Where there is a need to capture data on AEs the vignette methodology may be well suited to meet this need until other sources of data become available.

In conclusion, this study provides utility data for some rare AEs of biologic treatment and surgery-related complications in CD and demonstrate the value UK society places on avoiding AEs such as lymphoma and surgical complications such as anastomotic leak. These data fill a gap in the current literature and can be used in economic evaluations of new treatments.

## Supplementary Information



## Figures and Tables

**Table 1:** Socio-demographic Profile of the Sample

Participant Characteristics		Current sample (N=100)	ONS data 2011^21^
Age Mean (SD)		36.4 (11.7)	38.2
Gender Male n (%)		39 (39.0%)	49%
Main activity	Employed full time	73 (73%)	
	Employed part time	10 (10%)	
	Self employed	6 (6.0%)	
	Student	0 (0.0%)	1.9%
	Seeking work	1 (1.0%)	
	Long term sick leave	0 (0.0%)	
	Stay at home	2 (2.0%)	4.6%
	Retired	7 (7.0%)	9.8%
	Other	1 (1.0%)	
	Prefer not to answer	0 (0.0%)	
Education	No formal qualification	1 (1.0%)	
	Education till 16 years of age (GCSE or equivalent)	3 (3.0%)	
	Education till 18 years of age (GCSE or equivalent)	15 (15.0%)	
	Vocational/work based qualifications	3 (3.0%)	
	University degree	52 (52.0%)	
	Post graduate degree	26 (26.0%)	
	Other	0 (0.0%)	
EQ VAS Mean (SD)		84.84 (11.32)	
EQ-5D Index score		0.93 (0.16)	

**Table 2:** Mean TTO Scores from Reference State (Moderate to Severe CD) for all Health States (Best to Worst)

Health States	TTO Utility Score (mean, SD)	95% CIs	Disutility from Reference State ((mean, SD))
**Moderate to Severe CD (reference state)**	**0.70 (0.28)**	**0.65 – 0.75**	
Hypersensitivity	0.76 (0.19)	0.73 – 0.80	0* (0.18)
Injection site reactions	0.70 (0.24)	0.65 – 0.75	0 (0.22)
Serious infection	0.63 (0.24)	0.58 – 0.67	−0.07 (0.16)
Tuberculosis	0.47 (0.85)	0.34 – 0.66	−0.23 (0.8)
Lymphoma	0.44 (0.37)	0.37 – 0.52	−0.26 (0.29)
**Bowel surgery (reference state)**	**0.69 (0.28)**	**0.63 – 0.74**	
Wound infection	0.67 (0.28)	0.61 – 0.72	−0.02 (0.27)
Prolonged ileus / bowel obstruction	0.57 (0.38)	0.50 – 0.64	−0.11 (0.29)
Intra-abdominal abscess	0.56 (0.34)	0.49 – 0.62	−0.13 (0.25)
Anastomotic leak	0.48 (0.38)	0.40 – 0.55	−0.21 (0.27)

* This was set to 0 as it was valued higher than moderate to severe CD base state.
