# Supplementary material for: Disutility Study for Adult Patients with Moderate to Severe Crohn’s Disease
Source: J Health Econ Outcomes Res. 2019 Mar 26;6(2):47–60. doi: 10.36469/9685 (PMC7299447; doi:10.36469/9685)
Supplement: Supplementary file 1 [file jheor-6-2-9685-s01.pdf]

JHEOR Journal of Health Economics and Outcomes Research

OPEN ACCESS

Supplementary Online Content

Worbes-Cerezo M, Nafees B, Lloyd A. Disutility study for adult patients with moderate to severe Crohn's disease. *JHEOR*. 2019;6(2):47-60

Appendix 1: Final Health States

This supplementary material has been provided by the authors to give readers additional information about their work.

Appendix 1: Final Health States

Moderate to Severe CD (Reference Health State)

- You have a bowel condition which has caused inflammation around the bowel and digestive tract. You visit the doctor regularly so he/she can monitor your condition. You take medication regularly for your condition.
- You experience diarrhea several times a month and it lasts 1-2 days every time. You need to go to the toilet constantly all day when it happens and may have to take the day off from work. You experience severe abdominal cramps frequently. You have a lot of difficulties eating and have lost some amount of weight. You feel tired a lot in the day.
- You may need surgery to manage your disease.
- You are unable to do some of your usual activities such as work, housework, and shopping. You may have difficulties sleeping.
- You sometimes go out and visit family and friends.
- You sometimes feel depressed and anxious. You worry about how to manage your condition long-term.

Bowel Surgery for CD (Reference Health State)

- You have a bowel condition which has caused inflammation around the bowel and digestive tract. You visit the doctor regularly so he/she can monitor your condition. You take medication regularly for your condition.
- You experience diarrhea several times a month and it lasts 1-2 days every time. You need to go to the toilet constantly all day when it happens and may have to take the day off from work. You experience severe abdominal cramps frequently. You have a lot of difficulties eating and have lost some amount of weight. You feel tired a lot in the day.
- You recently had surgery and had to stay in hospital for around 5-7 days. After 5- 7 days, your doctor told you can go home and recovery will take up to 6-8 weeks. You experienced moderate pain or discomfort for several days after surgery. You needed to take pain killers while you were in the hospital.
- You were unable to do your usual activities while you were in hospital. You needed some help washing and dressing yourself. After you went home, you slowly resumed your normal activities however you were not able to lift anything heavy or do any strenuous activities such as exercise during the recovery or longer. After your recovery period you were able do your usual activities as normal.
- Friends and family came to visit you in hospital. You were unable to go out and visit family and friends. After recovery, you can go out to meet family and friends.
- You felt depressed and anxious whilst you were in hospital and worry that you may need surgery again. You worry about how to manage your condition long- term.

Seriuos Infection (Moderate to Severe)

- You have a bowel condition which has caused inflammation around the bowel and digestive tract. You visit the doctor regularly so he/she can monitor your condition. You take medication regularly for your condition.
- You experience diarrhea several times a month and it lasts 1-2 days every time. You need to go to the toilet constantly all day when it happens and may have to take the day off from work. You experience severe abdominal cramps frequently. You have a lot of difficulties eating and have lost some amount of weight. You feel tired a lot in the day.
- Last week, you experienced a serious infection due to your medication. This required hospitalization for a period of 3-5 days for intravenous antibiotic treatment. You felt more tired than usual and had to take some time off from work. You are at risk of this happening again.
- Normally you are a little restricted to do your usual daily activities such as work, housework or shopping. When you had the infection, you had problems with mobility and were in bed most of the day.

- Normally you are a little restricted to do your usual daily activities such as work, housework or shopping. When you had the infection, you had problems with mobility and were in bed most of the day.
- You can sometimes go out and visit family and friends. You were unable to do this when you had the infection.
- You are anxious and depressed. You worry about your condition.

Lymphoma (Moderate to Severe)

- You have a bowel condition which has caused inflammation around the bowel and digestive tract. You visit the doctor regularly so he/she can monitor your condition. You take medication regularly for your condition. You experience diarrhea several times a month and it lasts 1-2 days every time. You need to go to the toilet constantly all day when it happens and may have to take the day off from work. You experience severe abdominal cramps frequently. You have a lot of difficulties eating and have lost some amount of weight. You feel tired a lot in the day.
- You have developed a life-threatening complication of treatment which affects your lymph nodes. You have to undergo treatment for this which involves receiving regular cycles of treatment and regular hospital visits. You have lost your appetite. The treatment causes side effects such as vomiting, diarrhea, infections and hair loss.
- You are not able to do some of your usual daily activities such as work, or shopping. You may have problems with washing and dressing yourself on the days of your treatment.
- You can sometimes visit family and friends but prefer that they visit you.
- You are very anxious and depressed. You worry about your condition.

Injection Site Reactions (Moderate to Severe)

- You have a bowel condition which has caused inflammation around the bowel and digestive tract. You visit the doctor regularly so he/she can monitor your condition. You receive injection and other medication regularly for your condition.
- You experience diarrhea several times a month and it lasts 1-2 days every time. You need to go to the toilet constantly all day when it happens and may have to take the day off from work. You experience severe abdominal cramps frequently and are fine the rest of the time. You have a lot of difficulties eating and have lost some amount of weight. You feel tired a lot in the day.
- You experience a rash at the injection site every time you receive an injection for your treatment. Your skin becomes red, sometimes painful and uncomfortable or sore after the injection. This lasts several days.
- You are able to do your usual daily activities such as work, housework or shopping.
- You do not want to go out much when you have the rash. Generally you are able to go out and visit family and friends.
- You feel anxious and depressed about your next injection. You worry about your condition.

Wound Infection (Surgery)

- You have a bowel condition which has caused inflammation around the bowel and digestive tract. You visit the doctor regularly so he/she can monitor your condition. You take medication regularly for your condition.
- You experience diarrhea several times a month and it lasts 1-2 days every time. You need to go to the toilet constantly all day when it happens and may have to take the day off from work. You experience severe abdominal cramps frequently and are fine the rest of the time. You have a lot of difficulties eating and have lost some amount of weight. You feel tired a lot in the day.
- You recently had surgery after which you developed an infection at the site. The infection was painful and caused redness around the abdomen area. You had to stay in hospital for 3-5 days longer due to the

infection. You had to take antibiotics for it for 2 weeks. You have been asked to take it easy when you go home.

- At home you are unable to do your usual activities like housework and shopping for several weeks after the infection. When you came home from hospital you needed help in washing and dressing yourself.
- You sometimes go out and visit family and friends.
- You feel depressed and anxious whilst you are in hospital. You worry about your condition long-term.

Intra-abdominal Abcess (Surgery)

- You have a bowel condition which has caused inflammation around the bowel and digestive tract. You visit the doctor regularly so he/she can monitor your condition. You take medication regularly for your condition.
- You experience diarrhea several times a month and it lasts 1-2 days every time. You need to go to the toilet constantly all day when it happens and may have to take the day off from work. You experience severe abdominal cramps frequently. You feel tired a lot in the day.
- Following surgery you developed a mass in your abdomen and you had to stay in hospital for up to a week longer due to the abscess. The mass was painful and made you feel very poorly. You were unable to eat and drink as usual and experienced some weight loss. You had to take intravenous antibiotics to treat it. You were asked to take it easy when you went home and it is possible that you may need further surgery.
- At home you are unable to do your usual activities like housework and shopping for several weeks after the infection.
- You don't go out and visit family and friends.
- You felt depressed and anxious whilst you are in hospital. You worry about your condition getting worse.

Anastomatic Leak (Surgery)

- You have a bowel condition which has caused inflammation around the bowel and digestive tract. You visit the doctor regularly so he/she can monitor your condition. You take medication regularly for your condition.
- You experience diarrhea several times a month and it lasts 1-2 days every time. You need to go to the toilet constantly all day when it happens and may have to take the day off from work. You experience severe abdominal cramps frequently. You feel tired a lot in the day. You also experience nausea and vomiting frequently.
- You recently experienced surgery and had leakage from the intestinal wall into the body. This caused you to stay in hospital for several days after which you recovered at home. During your hospital stay you also had fever, which lasted for a few days. You were in severe pain in the abdomen and sometimes experienced nausea and vomiting. This usually stays in for a week. You were asked to take it easy when you went home and it is possible that you may need further surgery which may involve a temporary colostomy bag.
- At home you are unable to do your usual activities like housework and shopping for several weeks after the leak.
- You don't go out and visit family and friends.
- You felt depressed and anxious whilst you are in hospital. You worry about your condition getting worse.

Tuberculosis (Moderate to Severe)

- You have a bowel condition which has caused inflammation around the bowel and digestive tract. You visit the doctor regularly so he/she can monitor your condition. You take medication regularly for your condition.
- You experience diarrhea several times a month and it lasts 1-2 days every time. You need to go to the toilet constantly all day when it happens and may have to take the day off from work. You experience severe abdominal cramps frequently. You have a lot of difficulties eating and have lost some amount of weight. You feel tired a lot in the day.

- Recently, you developed a life-threatening infection in your lungs. You require careful monitoring and had to stay in hospital and receive treatment. You are at risk of infections and infecting others. You experienced high fever, cough, severe fatigue and you lost some weight. After you went home, you still have to come to hospital regularly for 3-6 months for monitoring and treatment. You will have to take multiple daily medications for this condition.
- At home you are unable to do your usual activities like housework and shopping for several weeks until your infection gets better.
- You don't go out and visit family and friends due to risk of infection.
- You feel depressed and anxious whilst you are in hospital. You worry about your condition getting worse.

Hypersensitivity (Moderate to Severe)

- You have a bowel condition which has caused inflammation around the bowel and digestive tract. You visit the doctor regularly so he/she can monitor your condition. You take medication regularly for your condition.
- You experience diarrhea several times a month and it lasts 1-2 days every time. You need to go to the toilet constantly all day when it happens and may have to take the day off from work. You experience severe abdominal cramps frequently. You have a lot of difficulties eating and have lost some amount of weight. You feel tired a lot in the day.
- You experienced a reaction from your treatment which resulted in shortness of breath, dizziness and rash and itching. This lasted for several days and you needed treatment for this.
- You are able to do activities like housework and shopping as usual.
- You didn't want to go out and visit family and friends when you had the rash. After treatment, you go out as usual.
- You sometimes worry about your condition.

Prolonged Ileus/Bowel Obstruction(Surgery)

- You have a bowel condition which has caused inflammation around the bowel and digestive tract. You visit the doctor regularly so he/she can monitor your condition. You take medication regularly for your condition.
- You experience diarrhea several times a month and it lasts 1-2 days every time. You need to go to the toilet constantly all day when it happens and may have to take the day off from work. You experience severe abdominal cramps frequently. You have a lot of difficulties eating and have lost some amount of weight. You feel tired a lot in the day. You also experience high fever and nausea and vomiting frequently.
- You recently had surgery after which you developed a blockage in your intestines and were hospitalized for further 3-5 days. You were unable to eat or drink and had fluids through a tube into your stomach. You were on antibiotics for a week and this delayed your recovery.
- You were unable to do your usual activities while you are in hospital. You may have needed some help washing and dressing yourself. After you went home, you slowly resumed your normal activities however you were not be able to lift anything heavy or do any strenuous activities such as exercise during the recovery or longer.
- You go out and visit family and friends.
- You felt depressed and anxious whilst you are in hospital. You worry a lot about your recovery and if your condition will get worse.
